# Electrochemically Deposited NiO Films as a Blocking Layer in *p*-Type Dye-Sensitized Solar Cells with an Impressive 45% Fill Factor

**DOI:** 10.3390/nano10010167

**Published:** 2020-01-17

**Authors:** Matteo Bonomo, Diego Di Girolamo, Marco Piccinni, Denis P. Dowling, Danilo Dini

**Affiliations:** 1 Department of Chemistry, University of Rome LA SAPIENZA, p.le Aldo Moro 5, 00185 Rome, Italy; matteo.bonomo@unito.it (M.B.); diego.digirolamo@uniroma1.it (D.D.G.); marcopiccinni90@gmail.com (M.P.); 2 Department of Chemistry and NIS Interdepartmental Centre and INSTM Reference Centre, University of Turin, via Pietro Giuria 7, 10125 Turin, Italy; 3 School of Mechanical & Materials Engineering, University College Dublin (UCD), Belfield, Dublin 4, Ireland; denis.dowling@ucd.ie

**Keywords:** blocking layer, electro-deposition, *p*-type DSSC, recombination reactions, optimized fill factor

## Abstract

The enhancement of photoelectrochemical conversion efficiency of *p*-type dye-sensitized solar cells (*p*-DSSCs) is necessary to build up effective tandem devices in which both anode and cathode are photoactive. The efficiency of a *p*-type device (2.5%) is roughly one order of magnitude lower than the *n*-type counterparts (13.1%), thus limiting the overall efficiency of the tandem cell, especially in terms of powered current density. This is mainly due to the recombination reaction that occurs especially at the photocathode (or Indium-doped Tin Oxide (ITO))/electrolyte interface. To minimize this phenomenon, a widely employed strategy is to deposit a compact film of NiO (acting as a blocking electrode) beneath the porous electrode. Here, we propose electrodeposition as a cheap, easy scalable and environmental-friendly approach to deposit nanometric films directly on ITO glass. The results are compared to a blocking layer made by means of sol-gel technique. Cells embodying a blocking layer substantially outperformed the reference device. Among them, BL_1.10V shows the best photoconversion efficiency (0.166%) and one of the highest values of fill factor (approaching 46%) ever reported. This is mainly due to an optimized surface roughness of the blocking layer assuring a good deposition of the porous layer. The effectiveness of the implementation of the blocking layer is further proved by means of Electrochemical Impedance Spectroscopy.

## 1. Introduction

Recently, a partial slowdown on research regarding Dye-Sensitized Solar Cells (DSSCs) has been evidenced. Notwithstanding this, DSSCs have some irreplaceable features, such as the use of environmentally-friendly materials [1], stability once exposed to solar radiation or oxygen [2], the possible obtainment of quasi-transparent devices [3], very good performance under low irradiance [4], among others [1]. DSSCs are usually made by sandwiching a photoactive electrode (i.e., anode) and a counter-electrode (CE). The latter should show a good catalytic activity toward the regeneration of a liquid [5] or quasi-solid electrolyte [6]. This configuration has two main drawbacks: on one hand, it involves the use of platinum, as a stable and efficient catalyst, which seriously undermines the sustainability of the devices [7]; on the other hand, just one of the electrodes is actively involved in light conversion. Interestingly, both these drawbacks could be overcome at once through the implementation of a photoactive counter-electrode, based on a sensitized *p*-type semiconductor [8,9]. The most widely employed semiconductor in *p*-type DSSCs is nickel oxide (NiO) [10], although some attempts have been also made with CuO [11], Cu_2_O [12,13] and other composite materials [14,15,16,17]. It is worth mentioning that *n*-type (i.e., TiO_2_-based) devices have been thoroughly investigated since their first appearance in 1991 [18], while Grätzel and collaborators reached a remarkable 13% of photo conversion efficiency (PCE) in 2014 [19]. Nevertheless, the attainment of efficient tandem devices is undermined by the relatively low performances of *p*-type counterparts in term of both current density and PCE [20]. This is mainly due to the occurrence of recombination phenomena [21]. As a matter of fact, the matching of the current density values powered by both photoanode and photocathode is needed to obtain effective tandem devices [22].

Recently, different approaches have been exploited to enhance the performances of NiO-based DSSCs. These consist of: (i) synthesis of sensitizers specifically designed for their application in p-DSSCs [20,23,24,25,26,27,28,29,30,31] and having appropriate photophysical features [32,33,34] to improve J_SC_; (ii) the engineering of iodine-based electrolyte [35] or its replacement with alternative redox couples [20,36,37,38,39] to obtain higher V_OC_; (iii) the amelioration of photocathodes electronic and morphological features [40,41,42,43]. Apart from these, the implementation of an insulating compact layer beneath the photocathode was proven to be an effective approach to reduce the rate of recombination reactions occurring at the FTO-coated glass/photocathode interface, acting as a charge blocking layer [44,45]. Moreover, it is also effective for obtaining a better contact between the substrate and the semiconducting film. This compact layer of a nanometer size can be deposited by means of different techniques such as Atomic Layer Deposition (ALD) [46,47], spray-pyrolysis (SP) [48], sol-gel approach (SG) [49], sputtering [50], among others. In the present case, we chose electrodeposition, which is an industrially attractive route due to having low costs and a scale-up capability [51]. Another advantage of electrodeposition is its versatility due to the possibility of controlling several process parameters (electrolyte composition, substrate, temperature, plating potential), which enable a strict control over the electrical and morphological quality of the resulting film. Different to the electrodeposition of organic materials with high redox potential [52], in the case of transition metal oxides, such as NiO, electrodeposition allowed us to work with aqueous electrolyte, thus minimizing the employment of toxic and hazardous organic solvents. 

**Scheme 1:**
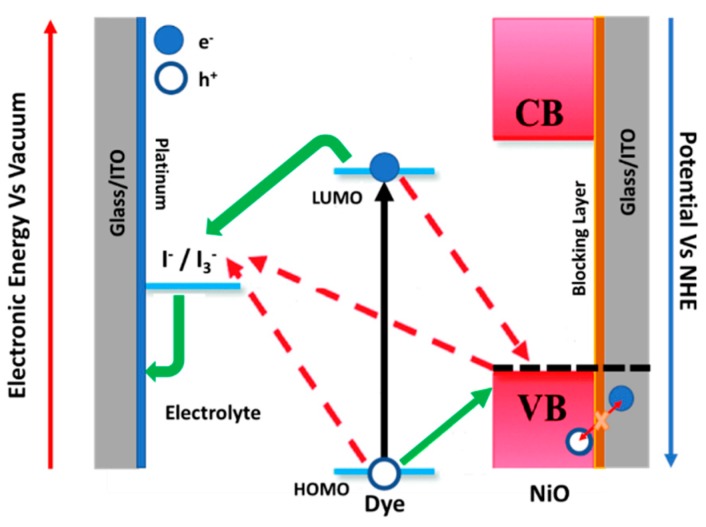
Scheme of the electronic process occurring in a p-DSC. The black line corresponds to the light-induced photoexcitation; green lines schematize the electrons (and holes) in an ideal device. Red lines accounts for undesired recombination reactions. The implementation of a blocking layer minimizes the recombination between the holes in the NiO VB and the electrons in the ITO layer.

Throughout the present paper, we present the electrochemical deposition of nanosized films of nickel oxide (NiO) to be implemented as a blocking layer in *p*-type Dye-Sensitized Solar Cells (Scheme 1). Different deposition potentials were scanned and the performance of blocking layers were deeply investigated. Once implemented in a complete device, the compact layer electrodeposited at 1.10 V clearly outperformed the others, leading to a PCE higher than 0.16% and to a remarkable fill factor close to 46%. These results account for the minimization of the TCO/NiO recombination reactions as evaluated through the technique of Electrochemical Impedance Spectroscopy (EIS) [53].

## 2. Experimental

NiO Nanoparticles (diameter < 35 nm) were purchased by US Research Nanomaterial Inc (Houston, TX, USA).; all the solvents and reactants were purchased from Sigma-Aldrich (St. Louis, MO, USA) at the highest degree of purity available and they were used without any further purification. P1 were synthetized in line with literature [54] and employed as a sensitizer. A 0.13 M nickel acetate (Alfa Aesar, Haverhill, MA, USA) solution was employed as an electrolyte for the NiO electrodeposition. Electrodeposition was carried out with an AUTOLAB PGSTAT12 from Metrohm (Herisau, Switzerland) potentiostat equipped with NOVA software. Ag/AgCl reference electrode and Pt wire as counter electrode were employed in a three-electrode configuration. The working electrode was vertically oriented and dipped in the solution, except for a small portion needed for electrical contact. After the electrodeposition, NiOOH films were rinsed with DI water to remove residues from the electrolyte. NiO films were obtained by thermal annealing in oven at 300 °C for 1 h. For sol-gel spin coated NiO, we adopted the procedure reported elsewhere [55]. NiO photocathodes were deposited onto pristine or modified ITO by spray-deposition. NiO nanoparticles were dispersed in methanol and sonicated in an ultrasonic bath to favor their homogenous dispersion. The resulting suspension is stable up to one week, then NPs tend to precipitate. Before each deposition, the suspension was sonicated for 30 min. The suspension was pumped into a nozzle (Ari Mist HP from Burgener Research International, Mississauga, ON, Canada) where a gaseous atomizer (i.e., N_2_) assured the formation micro-droplets that were simultaneously sprayed onto the (NiO-modified) ITO substrate. The latter was heated up to 65 °C to promote solvent vaporization. The movement of the nozzle (10 mm/s) could be controlled in both the *x* and *y* directions by using a software, whereas the distance between the nozzle and the substrate were set to 15 mm. The solution flow (0.01 mL/min) was controlled by an automatic syringe and the gas pressure was set to 552 Pa. The number of passes were thoughtfully chosen to obtain an electrode thickness of 2 μm. After being deposited, the electrodes were sintered in a conventional oven at 450 °C in order to assure the electric interconnection between the NiO NPs and to improve their adhesion to the substrate. The final product was a mesoporous NiO photocathode on top of a NiO compact layer.

Photocathodes were immersed (still warm) in a P1 solution (0.1 mM in absolute ethanol) [54]. The sensitization process was maintained overnight (i.e., 16 h). The electrode were then removed by using the dipping solution and abundantly washed with ethanol in order to remove the excess of not chemisorbed dye. The so obtained P1-sensitized photocathodes were sandwiched with a Pt counter-electrode [56,57] with a Surlyn mask aging as both sealant and spacer. The iodine-based electrolyte (i.e., HSE from Solaronix) was inserted in the device from a hole in the Surlyn mask by using the back-vacuum filling technique. The hole was then closed by a thermosetting bi-component commercial resin. 

The surface roughness of the electrode was estimated with an optical profilometer from Veeco. For the determination of the dye-loading, a sensitized electrode was immersed in a NaOH water solution (0.1 M) for 12 h. The obtained solution was spectroscopically characterized to determine the amount of dye desorbed from the photocathode through the use of a calibration curve-based method. Photoelectrochemical characterization consisting of the determination of the current-potential (*J*-*V*) curve by using the solar simulator (class A) at 1000 W m^−2^ with artificial solar spectrum AM 1.5 G. Electrochemical Impedance Spectroscopy (EIS) measurements were performed with an AUTOLAB PGSTAT12^®^ from Metrohm (Herisau, Switzerland) with the condition of open circuit potential under solar simulator illumination. In the EIS experiments the potential perturbation had an amplitude of 10^−2^ V. Impedance spectra were recorded within the frequency ranges 10^−1^−10^5^ Hz. Electrochemical impedance spectra were fitted using *Z*-View software from Scribner Associates Inc (Southern Pines, NC, USA). To interpolate the impedance spectra of NiO based *p*-DSCs, we employed a self-developed equivalent circuit [35] adapting the transmission line model first proposed by Bisquert et al. for the impedance analysis of TiO_2_-based *n*-DSCs [58].

## 3. Results and Discussion

**Figure 1:**
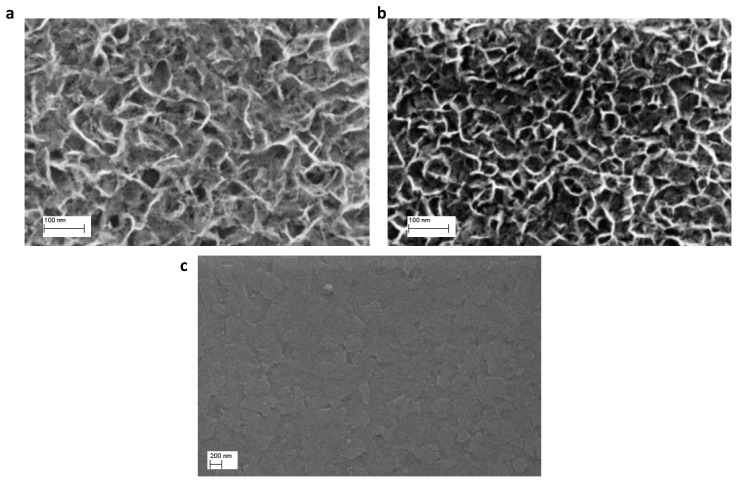
(**a**) Top view SEM micrograph of NiO deposited at 1.00 V vs. Ag/AgCl. (**b**) Top view SEM micrograph of NiO deposited at 1.10 V vs. Ag/AgCl. (**c**) Top view SEM micrograph of NiO deposited by sol-gel.

In our previous work we investigated in detail the anodic electrodeposition of NiO [59]. In potentiostatic condition above 1.00 V vs. Ag/AgCl, it is possible to obtain a full and homogenous coverage of the ITO electrode within minutes. The film deposited is constituted by a mixture of γ-β NiOOH which is converted to NiO with a thermal treatment at 300 °C [60]. Notably, the morphology of the electrodeposited films can be controlled by changing the plating potential. In fact, as shown in Figure 1, the surface porosity of the film deposited at 1.00 V vs. Ag/AgCl is higher than for the film deposited at 1.10 V vs. Ag/AgCl. The morphologies of different electrodeposited NiO films (1.00 V, 1.05 V and 1.10 V) have been photographed and thoroughly discussed in our previous publication [59]. In the present work focusing on DSSCs, we show SEM images in order to demonstrate that the surface porosity of NiO can be tuned upon changes of the electroplating potential. This finding indicates an attractive aspect of the use of the anodic electrodeposition route. The comparison of the morphologies of the films obtained at 1.00 V and 1.10 V makes clear how finely surface porosity can be tuned with an electrochemical method of deposition [61,62]. It must be noted that the thickness of the films is below 100 nm, which is one order of magnitude less than the mesoporous NiO films where the dye is anchored. Nonetheless, the surface porosity of compact NiO films has been shown to be crucial for the hole extraction from lead halide perovskites in perovskite solar cells [63,64]. It is therefore quite interesting to investigate whether this feature influences their behavior as blocking layers in p-DSSC as well. The synthesis conditions for the electrodeposited NiO blocking layers employed in this film are summarized in Table 1. 

It is worth mentioning that an extensive characterization of both NiOOH and NiO films fall outside the scope of the present work, which is mainly focused on the application of the electrodeposited films in DSSCs. XPS characterization has been already presented in one of our previous works [59]. We found that the small thickness of the resulting electrodeposited films of NiO, XRD, and Raman spectroscopy could not provide an unambiguous and detectable signal that is associated with such a layer. The crystallinity of NiO obtained via the electrodeposition route is investigated and discussed in the works by Wu [65,66].

**Figure 2:**
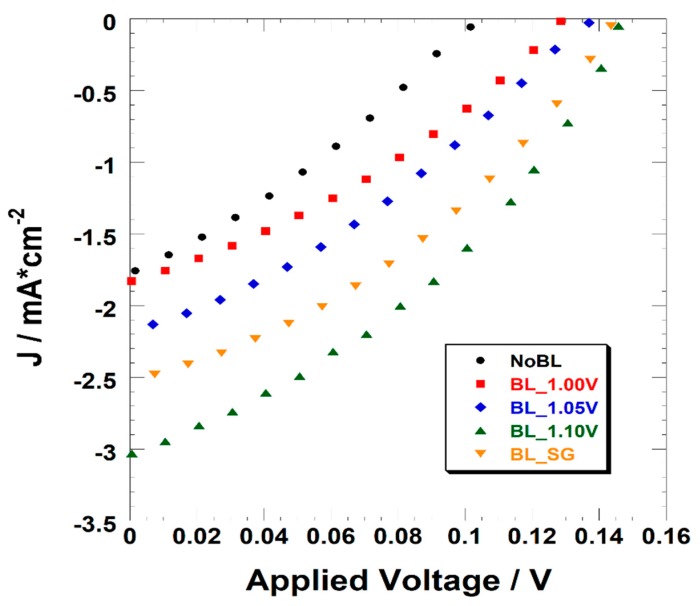
*J*-*V* Curves of Dye-Sensitized Solar Cells made with or without (black dots) a blocking layer. The latter is electrochemically deposited at 1.00 V (red squares), 1.05 V (blue diamonds), 1.10 V (green triangles), or deposited by sol-gel (orange triangles).

As one can see from Figure 2 and Table 2, the employment of a BL remarkably improved the photoelectrochemical performances of the NiO-based photocathodes. More in detail, the implementation of a compact blocking layer further enhanced the open circuit voltage of the cell. It is worth mentioning that the V_OC_ is a thermodynamic parameter depending on the energetic difference between the valence band (VB) of the *p*-type semiconductor (i.e., NiO) and the redox potential of the redox mediator. Nevertheless, it is strongly influenced by recombination phenomena that involve the injection of electrons in the VB of the NiO. Within the latter, the hole density is then reduced and, straightforwardly, the Fermi’s level of the semiconductor is upshifted [67,68]. This results in a lower V_OC_ compared to the thermodynamic value, thereby undermining the photoelectrochemical performances. These recombination reactions usually occur both at the electrode/electrolyte interface or at the ITO/electrode interface [69]. The insertion of a blocking layer minimized the recombination reaction at the latter interface. Actually, in the present case, the V_OC_ increases from 102.6 mV (**NoBL**) to 130.5, 136.9, and 150.6 when the blocking layer is deposited at 1.00, 1.05, and 1.10 V, respectively. The device embedding a blocking layer made by Sol-Gel uses a V_OC_ of 147.4 mV. 

Apart from the V_OC_, the effect of the blocking layer is also evident on the FF that, in case of **BL 1.10 V** reached an impressive value of 45% that, as far as we are aware, is one of the higher ever reported [10]. As already stated before, the implementation of a blocking layer beneath the photocathode leads to a reduction in the recombination phenomena. As a clear result of this, a higher number of injected holes could effectively reach the ITO-coated glass, resulting in a higher current density. Under these circumstances, dye-loading and current density values do not end up directly related as a result. In fact, the boost in J_SC_ is due to the implementation of a blocking layer (*vide infra*), which is the recombination phenomena at the ITO/electrode interface that is severely quenched. 

**Figure 3:**
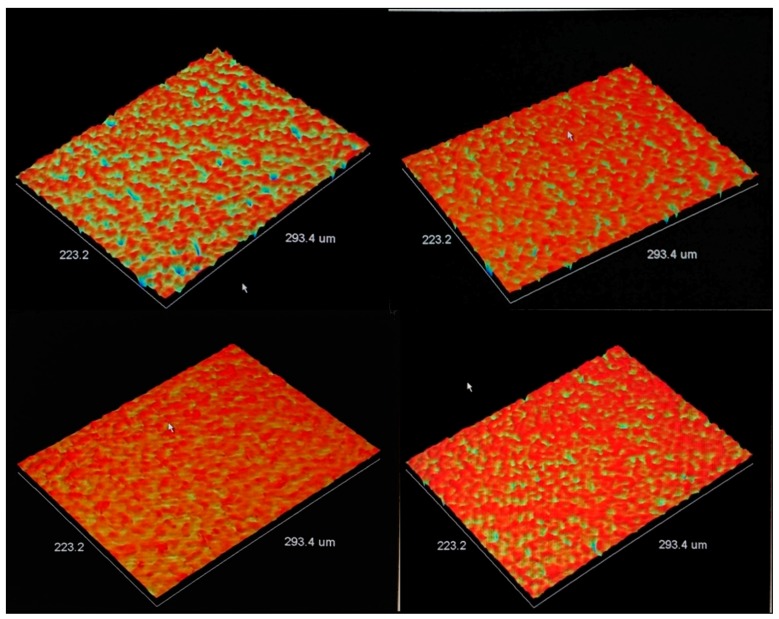
Investigation of the surface roughness of NiO photocathodes implementing a blocking layer electrochemically deposited at 1.00 V (top left), 1.05 V (top right), 1.10 V (bottom left) or deposited by Sol-Gel (bottom right).

The presence and the nature of the blocking layer seems also to deeply influence the morphology of the mesoporous NiO films, whereas the porosity is constant (Figure 3). As a matter of fact, the photocathode without the blocking layer reflects the low roughness of the ITO, whereas the implementation of an electrochemically deposited NiO compact layer increases the surface roughness up to 635, 493, and 250 nm for **BL 1.00 V**, **BL 1.05 V,** and **BL 1.10 V**, respectively (Table 2). As recently reported by some of us [59], this trend reflects the homogeneity in the ITO coating experimented by the film deposited at higher applied voltage. Dye loading seems to be poorly affected by the surface roughness. As a matter of fact, dye loading is mainly controlled by the porosity of the photoanodic substrate. It is worth mentioning that an excessive surface exposure to the electrolyte should be avoided in order to minimize the recombination reaction occurring between the iodide anions and the unprotected (by the anchored dye) Ni^3+^ surface sites. This could be one of the reasons behind the better figures of merit showed by **BL 1.10 V** and **BL Sol-Gel**, which, in turn, are the smoother electrodes. 

To further investigate the photoelectrochemical behavior of the photocathodes, we decided to use Electrochemical Impedance Spectroscopy. EIS is a very powerful tool for investigating the charge transport and transfer phenomena occurring within the photocathode or at the TCO/semiconductor and semiconductor/electrolyte interfaces. To interpolate experimental data, we employed a custom-made equivalent circuit that consisted of (i) a resistor (R_s_); (ii) a parallel-connected resistor/capacitor element (R_CE_ and CPE_CE_) and (iii) a Transmission Line element (TL) [35,70]. In *p*-type DSSCs, two semicircles could be evidenced. The first, at a relatively high applied frequency, accounts for the charge transfer reaction occurring at the counter-electrode (i.e., the Pt-catalyzed reduction of triodiode into iodide and it is modeled by the R_CE_/CPE_CE_ element), whereas the second is related to the charge transfer phenomena occurring at the electrode/electrolyte interface and it could be easily described by the TL element [71]. Some authors evidenced that the first semicircle arises from the convolution of two (smaller) semicircles that describe two different phenomena but occurring at a similar time-scale, namely the charge transfer at the electrolyte/CE interface and the charge transfer at the TCO/NiO interface [45]. Two examples of evidence support such a hypothesis: (i) R_CE_ is much higher compared to the one obtained in a symmetrical cell, in which the electrolyte is sandwiched between two platinum electrodes; (ii) the photocathode deposition technique influences the size of the first semicircle, as the latter is sensibly lower for spray-deposited electrode with respect to screen-printed ones. As a matter of fact, spray deposition assures a better coverage of the TCO substrate, thereby lowering the interfacial resistance. 

**Figure 4:**
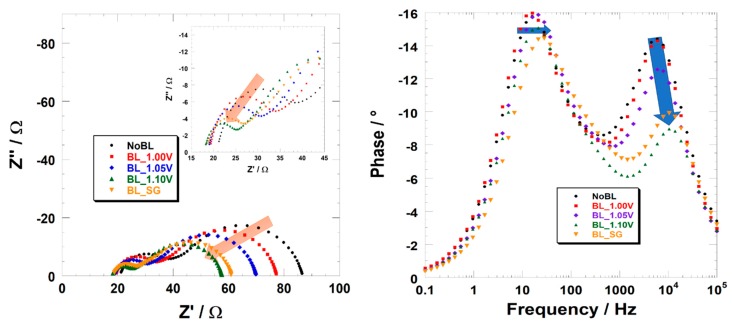
Electrochemical Impedance spectra of Dye-Sensitized Solar Cells made with or without (black dots) a blocking layer. The latter is electrochemically deposited at 1.00 V (red squares), 1.05 V (blue diamonds), 1.10 V (green triangles), or deposited by sol-gel (orange triangles). EIS spectra are reported in both the Nyquist (on the left) and Bode (on the right) representation. The insert is a zoom of the high-frequency domain to highlight the downsizing of the first semicircle.

The EIS spectra of our devices are reported in Figure 4 (Nyquist and Bode’s representation, left and right, respectively). The parameters extracted by the interpolation of experimental data are summarized in Table 3. EIS spectra of the device with a blocking layer are smaller compared to **NoBL**, accounting for a less resistive behavior of the photocathode. Actually, the charge recombination resistance (i.e., the diameter of the second semicircle, R_rec_) also decreases following on from the implementation of the blocking layer; the lower R_rec_ the more probable the recombination reactions. Notwithstanding this apparent inconsistence with respect to JV data, the effectiveness of the charge transport throughout the electrode could be estimated by the evaluation of L_h_ (namely the hole diffusion length) that could be calculated from L_h_ = l*(R_rec_/R_t_)^1/2^, with l being the nominal thickness of the NiO film (2 μm) and R_t_ being the resistance at the hole diffusion throughout the photoanode. L_h_ is 3.65 μm, 4.50 μm, 4.48 μm, 4.30 μm, and 3.88 μm for **NoBL**, **BL 1.00 V**, **BL 1.05 V**, **BL 1.10 V,** and **BL Sol-Gel,** respectively. The quite similar values of L_h_ evidences how the implementation of a blocking layer beneath the NiO photocathode just slightly influences the recombination phenomena occurring at the electrolyte interface. This is further confirmed by the slight shift of the low-frequency peak in the Bode’s plot whose maximum is related to the timescale of the recombination phenomena. 

The clearest effect of the presence of the blocking layer is the reduction of the magnitude of the high-frequencies semicircle. Following on from the interpretation of Ho et al. [45], the reduction could be solely ascribed to a diminution in the resistance at the TCO/NiO interface being the counter-electrode/electrolyte interface (i.e., the anodic interface of our device) unaffected by the presence of a blocking layer at the cathode. Remarkably, the first semicircle resistance is 5.2 Ω and 5.9 Ω for **BL 1.10 V** and **BL Sol-Gel**, respectively. This corresponds to being 2.5 times lower than the one experimented on by the device **NoBL** (13.5 Ω). In correspondence of the uncoated counter-electrode/electrolyte with resistance close to 5 Ω (as proved by electrochemical impedance analyses of symmetrical cells) the TCO/NiO interfacial resistance becomes negligible if an optimized blocking layer is employed. On the other hand, such a parameter increases up to ca. 10 Ω when the blocking layer is absent. It is worth mentioning, that the series resistance values could have an effect on the estimation of these parameters. Yet, in our case R_S_ value is about the same for all the investigated devices. 

As further proof of that, Bode’s plots show how the maximum of the high-frequency peak (associated with the first semicircle in the Nyquist’s plot) clearly shows a shift toward higher applied frequencies if the devices present the blocking layer. The characteristic time measured for **BL 1.10 V** (i.e., 21 μs) is consistent with the charge transfer catalyzed by the Pt electrode. On the other hand, the corresponding value for **NoBL** is 96 μs, which is extremely long for a CT reaction. Therefore, an additional event occurring at a similar timescale (and straightforwardly indiscernible by the interpolation procedure) should be considered. This is the ITO/NiO charge transfer. To further prove this hypotheses, it could be very useful to perform an EIS experiment at a different light intensity; as a matter of fact, the charge transfer kinetic at the counter-electrode should not be influenced by the degree of illumination. On the other hand, at low light intensity, ITO/NiO should became negligible for all the electrodes (no matter the presence and the nature of the blocking layer) because of the reduced number of photo-injected charges. This will definitively prove the double contribution to the high-frequency peak. Unfortunately, at the moment, we have no evidence for this type of instrumentation. However, we are quite confident that the experimental data showed here above are enough to prove the effectiveness of the implementation of our blocking layers.

## 4. Conclusions 

Throughout the present paper, we report on the electrochemical deposition of nanometric compact films of nickel oxide to be used as a blocking layer in *p*-type DSSCs. Once implemented in a complete device, blocking layers allow us to substantially enhance the photoconversion efficiency if compared to a reference cell. The film deposited at 1.10 V (vs. Ag/AgCl) shows the best photoelectrochemical performances (V_OC_ = 150.6 mV; J_SC_ = −3.08 mA·cm^−2^; PCE = 0.166%) consisting in a 400-fold increase compared to the device without any blocking layer. More remarkably, it exhibits an FF value as high as 46%, which is one of the highest ever reported in the literature. This is mainly due to an optimized surface roughness and thickness that allow the growth of a homogenously porous electrode above the compact layer. Indeed, an excessively thin film is not able to effectively reduce the recombination reaction at the ITO/electrolyte whereas, on the other hand, a thick film leads to sizeable transport resistance. Furthermore, we take advantage of electrochemical impedance spectroscopy (EIS) to investigate the effect of the presence of the blocking layer on the (photo) electrochemical properties of the devices. A clear shift of the high-frequency peak (Bode’s representation) toward higher frequencies is evidenced. In practice, the ITO/electrolyte interface becomes negligible. We are confident that the findings reported throughout the present manuscript are an interesting starting point to produce low-cost and environmentally-friendly photocathode that can be mainly implemented in tandem DSSCs.

## Figures and Tables

**Table 1:** The different NiO films produced and discussed in this work.

NAME	Potential V vs. Ag/AgCl	Time s	Reduction
*BL 1.00 V*	1.00	900	No
*BL 1.05 V*	1.05	300	No
*BL 1.10 V*	1.10	120	No
**sg-NiO**	Sol-gel spin coating from a NiCl_2_ solution [55]		

**Table 2:** Photoelectrochemical figures of merit of NiO-based DSSCs with or without the implementation of a blocking layer.

Sample	V_OC_/mV	J_SC_/mA·cm^−2^	FF/%	PCE/%	Roughness/nm	Dye Loading/ 10^8^·mmol·cm^−2^
*No Blocking Layer*	101.6 ± 1.6	−1.77 ± 0.26	30.7 ± 1.0	0.055 ± 0.003	176 ± 10	3.19 ± 0.29
*BL 1.00 V*	130.5 ± 2.1	−1.84 ± 0.15	33.2 ± 1.1	0.079 ± 0.006	635 ± 13	3.45 ± 0.26
*BL 1.05 V*	136.9 ± 2.0	−2.19 ± 0.14	35.8 ± 1.6	0.098 ± 0.010	493 ± 23	3.42 ± 0.24
*BL 1.10 V*	150.6 ± 1.8	−3.08 ± 0.23	45.9 ± 1.9	0.166 ± 0.013	250 ± 10	3.32 ± 0.23
*BL Sol-Gel*	147.4 ± 1.2	−2.53 ± 0.25	36.0 ± 0.9	0.134 ± 0.009	323 ± 14	3.23 ± 0.25

**Table 3:** Photoelectrochemical parameters obtained from the interpolation of the experimental point of EIS spectra.

	NoBL	BL 1.00 V	BL 1.05 V	BL 1.10 V	BL Sol-Gel
*R_s_*/Ω	20.5 ± 0.2	18.9 ± 0.3	18.6 ± 0.3	17.8 ± 0.6	18.2 ± 04
*R*_1_/Ω	13.5 ± 0.5	11.2 ± 0.4	8.6 ± 0.6	5.9 ± 0.3	5.2 ± 0.3
*C*_1_/μF	7.1 ± 0.3	4.9 ± 0.3	4.3 ± 0.1	3.5 ± 0.2	3.7 ± 0.2
*R_t_*/Ω	17.5 ± 0.6	15.0 ± 0.5	13.5 ± 0.6	11.7 ± 0.3	14.7 ± 0.8
*R_rec_*/Ω	85.4 ± 5.3	75.5 ± 3.6	68.5 ± 4.8	54.5 ± 3.9	55.5 ± 5.2
*C_μ_*/mF	0.34	0.39	0.35	0.36	0.38
*L_h_*/μm	3.65 ± 0.23	4.50 ± 0.28	4.48 ± 0.36	4.30 ± 0.43	3.88 ± 0.18
*t*_1_/μs	95.9 ± 3.3	54.9 ± 3.6	37.0 ± 2.8	20.7 ± 1.9	19.2 ± 1.2
*t*_2_/ms	29.1 ± 2.3	29.5 ± 1.2	24.0 ± 1.9	19.6 ± 0.5	21.1 ± 1.3
